# Clinical features and outcomes of pregnancies complicated by coexisting gestational diabetes and hypertensive disorders

**DOI:** 10.3389/fmed.2025.1656391

**Published:** 2025-10-22

**Authors:** Chaoying Jia, Lanying Bo, Shu Xiao, Shunlan Du

**Affiliations:** Obstetrical Department, Dongyang Hospital Affiliated to Wenzhou Medical University, Dongyang, Zhejiang, China

**Keywords:** gestational diabetes mellitus, pregnancy induced hypertension, adverse pregnancy outcome, clinical characteristics, metabolic predictors

## Abstract

**Background:**

Gestational diabetes mellitus (GDM) and hypertensive disorder complicating pregnancy (HDP) share pathophysiological mechanisms that increase the risk for adverse maternal and neonatal outcomes. However, their combined impact remains underexplored.

**Objective:**

To assess the clinical characteristics, therapeutic interventions, and maternal–fetal outcomes in pregnancies complicated by both GDM and HDP.

**Methods:**

A retrospective study was conducted involving 82 women with GDM complicated by HDP and 118 healthy pregnant controls. Clinical parameters, mode of delivery, and pregnancy outcomes were compared between groups. Logistic regression analysis was performed to identify independent predictors of GDM complicated by HDP.

**Results:**

Women with GDM complicated by HDP exhibited significantly higher pre-pregnancy weight, body mass index (BMI), fasting blood glucose, triglycerides, and high-density lipoprotein (HDL) cholesterol compared with healthy controls (*p* < 0.05). Logistic regression identified these variables as independent predictors of GDM + HDP (*p* < 0.001). The GDM + HDP group also had higher rates of cesarean delivery and adverse pregnancy outcomes. Individualized treatment targeting glycemic and blood pressure control significantly improved metabolic parameters and reduced the incidence of complications (*p* < 0.05).

**Conclusion:**

Pre-pregnancy metabolic factors, including BMI, fasting blood glucose, triglycerides, and HDL cholesterol, are strong predictors of GDM complicated by HDP. Early identification and individualized management of these high-risk pregnancies can effectively reduce adverse maternal and neonatal outcomes.

## 1 Introduction

Pregnancy is a special physiological period for women (1). Compared to non-pregnant women, pregnant women (PW) exhibit significantly elevated levels of dietary fat intake, fat absorption, hepatic lipid synthesis, and circulating insulin and leptin concentrations. These physiological metabolic changes are significant for maintaining the normal growth and development of the fetus (2, 3). However, persistent abnormal increases can lead to metabolic disorders in PW, such as hypertensive disorder complicating pregnancy (HDP) and gestational diabetes mellitus (GDM). These conditions elevate the risk of adverse pregnancy outcomes, including preterm delivery, macrosomia, cesarean section, polyhydramnios, and growth restriction, posing serious threats to maternal and fetal health (4–6). In addition, the risk of GDM in the second pregnancy is about 50%, and the risk of HDP is about 30% (7). In recent years, it has been gradually found that GDM and HDP exist simultaneously in some PW, which causes the abnormal rate of fetal growth and the adverse pregnancy outcome of newborns to be higher than that of a single complication (8). GDM and HDP during pregnancy have a higher risk of adverse outcomes, which is a risk factor for adverse pregnancy outcomes (9). Clinically, HDP and GDM share similar pathological mechanisms, such as endothelial dysfunction, dyslipidemia, micro-inflammatory reactions, and disorders of the renin-angiotensin-aldosterone system. These factors elevate the risk of HDP in women with GDM (10). Attention must be given to the safety of pregnancies complicated by GDM and HDP. Currently, research primarily focuses on single conditions—GDM or HDP—and their treatment, with limited consensus on the combined impact of these conditions. This study retrospectively analyzes the clinical characteristics and treatment of PW with GDM and HDP, assesses their effects, and offers medication guidance to inform clinical interventions and enhance maternal and fetal safety.

## 2 Methods

### 2.1 Study subjects

We retrospectively analyzed clinical data from 82 singleton PW diagnosed with GDM and HDP, as well as 118 healthy singleton PW, all of whom received routine prenatal care and delivered at our hospital between January 2022 and December 2023. These participants were categorized into a GDM group combined with an HDP group and a healthy control group (HCG).

### 2.2 Sample size estimation

According to the sample size estimation using G*Power software, the study requires at least 42 participants in the GDM combined with HDP group and 72 participants in the healthy control group to ensure 80% statistical power at a significance level of 0.05 for detecting a significant difference in the incidence of adverse pregnancy outcomes between the two groups. To compensate for potential sample loss, a final total of 82 participants in the GDM combined with HDP group and 118 participants in the healthy control group were included, meeting the statistical requirements.

### 2.3 Inclusion and exclusion criteria

Inclusion criteria for the GDM combined with the HDP group:

1. Fulfilling the diagnostic criteria for both GDM and HDP, which are as follows: FPG ≥ 5.1 mmol/L, 1-h blood glucose ≥ 10.0 mmol/L, 2-h blood glucose ≥ 8.5 mmol/L at 24–28 weeks of gestation, as determined by the oral glucose tolerance test (OGTT); GDM is diagnosed based on any abnormal values (11), and systolic blood pressure (SBP) ≥ 140 mmHg and/or diastolic blood pressure (DBP) ≥ 90 mmHg for the first time after 20 weeks of gestation, with negative urine protein test (12).
2. All patients had regular checkups and were delivered in this hospital.
3. All patients provide informed consent.

Exclusion criteria:

1. History of diabetes, hypertension, or other endocrine disorders before pregnancy.
2. Presence of other pregnancy-related conditions (e.g., cholestasis of pregnancy).
3. Active infectious diseases, including hepatitis B, hepatitis C, HIV, syphilis, tuberculosis, or other systemic infections.
4. Induced labor due to fetal malformation or intrauterine fetal death.
5. Chronic kidney disease, nephrotic syndrome, or other significant organ dysfunctions.
6. Poor compliance with follow-up appointments.

Inclusion criteria for the HCG:

1. All participants had live births.
2. All had regular prenatal checkups and were delivered at this hospital.
3. No abnormalities were found during pregnancy checkups.
4. All patients provided informed consent.

Exclusion criteria:

1. Presence of combined endocrine, immune, or blood system diseases.
2. Any form of organ dysfunction.
3. History of pre-existing chronic or systemic diseases.
4. Poor compliance with regular checkups.

The current study was approved by the Ethics Committee of the Dongyang Hospital Affiliated to Wenzhou Medical University (2024-YX-323). Written informed consents from all patients were obtained for any experimental work with humans. Exclusion criteria were strictly defined to ensure homogeneous groups and avoid biases related to co-existing conditions.

### 2.4 Data collection and observation indices

Clinical and laboratory data were retrospectively collected from the medical records of all participants under the supervision of experienced obstetricians and gynecologists. Maternal demographic and anthropometric information included age, pre-pregnancy weight, pre-delivery weight, gestational weight gain, height, and body mass index (BMI). Additional data included family history of diabetes and hypertension, parity, and educational level. Gestational weight gain was calculated as the difference between maternal weight at delivery and pre-pregnancy weight, which measures weight change during pregnancy.

Early pregnancy biochemical markers, including fasting blood glucose (FBG), postprandial blood glucose (PBG), glycosylated hemoglobin (HbA1c), total cholesterol (TC), triglycerides (TRI), high-density lipoprotein cholesterol (HDLC), and low-density lipoprotein cholesterol (LDLC), were obtained from routine prenatal laboratory assessments conducted at our hospital between 6 and 16 weeks of gestation. All PW underwent a 75-g OGTT between 24 and 28 weeks of gestation. Fasting venous blood (5 mL) was drawn in the morning after an overnight fast of at least 8 h. Blood samples were centrifuged at 3,000 rpm for 15 min, and serum was analyzed using fully automated biochemical analyzers according to standard laboratory protocols. PBG levels were measured following the same processing protocol after the participants consumed a standardized meal or glucose challenge, ensuring consistent assessment of glycemic control. Blood pressure measurements were recorded using automated sphygmomanometers to evaluate hypertension control, including systolic blood pressure (SBP) and diastolic blood pressure (DBP). Information on treatment interventions for GDM and HDP was also documented.

Delivery characteristics, including mode of delivery and postpartum hemorrhage volume, were collected. Adverse perinatal outcomes were recorded as binary variables (yes/no) and defined as follows: macrosomia (birth weight ≥ 4,000 g), low birth weight (<2,500 g), preterm delivery (<37 weeks gestation), oligohydramnios (amniotic fluid index < 5 cm), fetal growth restriction (estimated fetal weight <10th percentile for gestational age), premature rupture of membranes (rupture of membranes prior to the onset of labor), and postpartum hemorrhage (blood loss ≥500 mL for vaginal delivery or ≥1,000 mL for cesarean section).

These indices were analyzed to compare participants with GDM combined with HDP and healthy controls and assess the effects of treatment interventions on maternal and neonatal outcomes. The data collection and processing protocol ensured accuracy, consistency, and reliability for clinical and biochemical variables, minimizing potential biases due to data heterogeneity or measurement errors.

### 2.5 Therapeutic method

Clinical management of PW with GDM and HDP was conducted in accordance with standard clinical guidelines. Patients with GDM initially received lifestyle interventions, including dietary modification and physical activity. Insulin therapy (insulin aspart, D Novotel) was administered at doses ranging from 8 IU to 34 IU when glycemic targets were not achieved with lifestyle measures alone. For hypertension, patients were treated with labetalol hydrochloride (0.1–0.2 g, twice daily), and, when indicated, a combination of magnesium sulfate (30 mL, intravenous infusion) and nifedipine sustained-release tablets (10 mg) was administered.

Patients who received no pharmacological therapy but were managed with lifestyle interventions alone were classified as the “untreated group.” This group was distinct from the healthy control group, which included women without GDM or HDP. Treatment regimens for all patients were individualized according to clinical condition, with continuous monitoring of blood glucose and blood pressure to guide therapy. Comparisons were made between treated and untreated patients with GDM and HDP and between case and control groups to assess the impact of therapeutic interventions on maternal and perinatal outcomes.

### 2.6 Statistical methods

Prism 9.4.1 was used for image processing, while SPSS 23.0 was used to analyze data. The measurement data were represented by ( $\bar{x}\pm S$ ) and had a normal distribution with homogeneous variance. An independent sample t-test was used to compare the two groups, and a paired t-test was applied to compare the groups among themselves. The χ2 test was used to assess intergroup comparisons for categorical data, which was reported as [n(%)]. Univariate logistic regression analyses were initially performed to explore associations between individual variables and adverse pregnancy outcomes. Variables with a *p*-value < 0.10 in univariate analysis or those considered clinically significant based on prior evidence were included in the multivariate logistic regression model to identify independent predictors. *p*-values were considered statistically significant if they were less than 0.05.

### 2.7 Research flow chart

**Figure 1:**
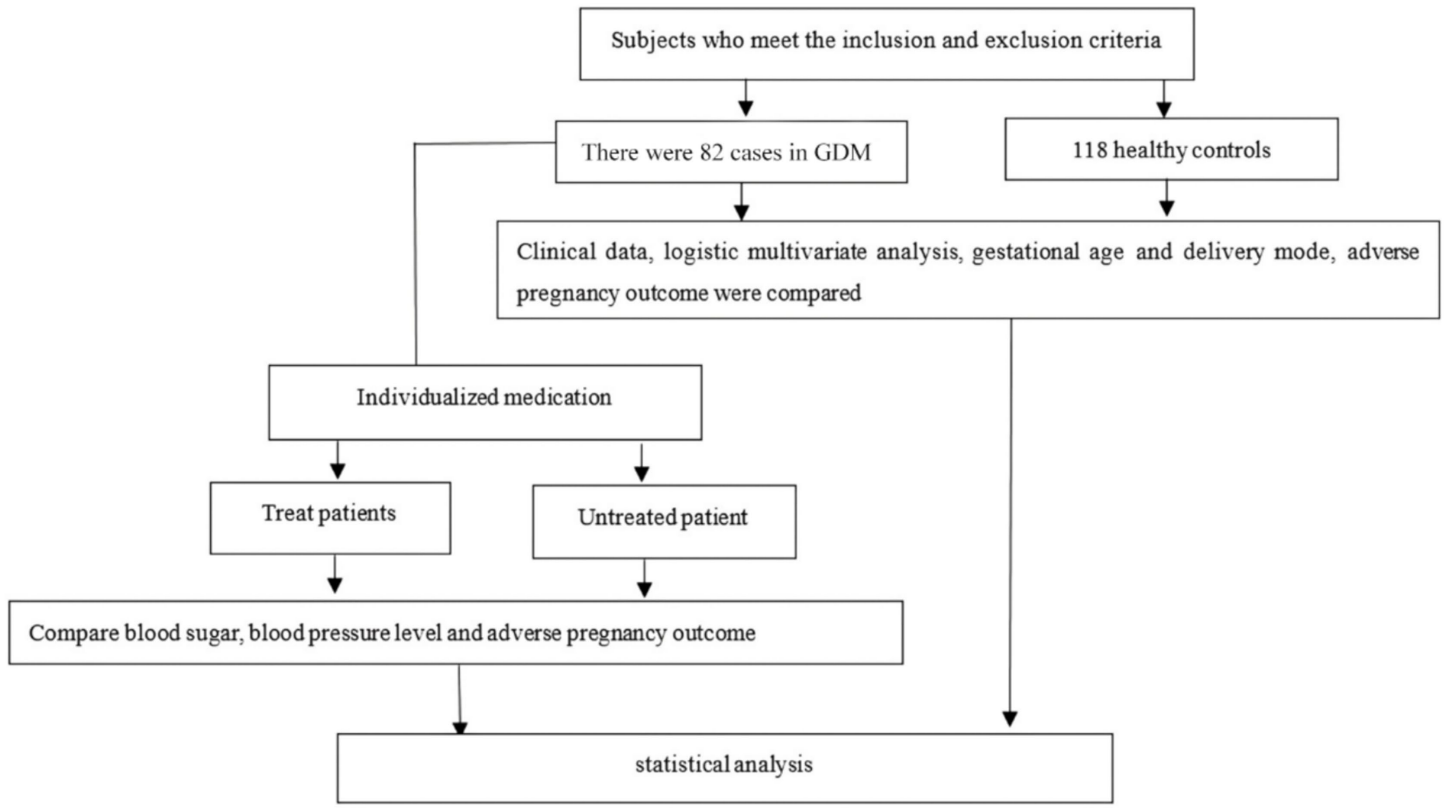
Research flow chart.

The overall design and participant selection process of this study are illustrated in Figure 1. The flow chart depicts the screening, inclusion, and exclusion of participants and the final allocation into the GDM combined with HDP group and the healthy control group, providing a clear overview of the study workflow.

## 3 Results

### 3.1 Clinical data between GDM combined with HDP group and HCG

No significant differences were observed between the two groups in terms of maternal height, educational level, LDLC, TC, or gestational weight gain (*p* > 0.05). Compared with the HCG, women in the GDM combined with HDP group exhibited significantly higher values during the first trimester for age, pre-pregnancy weight, pre-pregnancy body mass index (BMI), family history of hypertension and diabetes, parity, FBG, triglycerides, and HDLC (*p* < 0.05; Table 1).

**Table 1:** Comparison of clinical characteristics between Women with GDM complicated by HDP and the healthy control group (HCG).

Items	GDMplus HDP group (*n* = 82)	HCG (*n* = 118)	*t/χ^2^/Z*	*P*
Age	31.69 ± 5.03	29.96 ± 4.35	2.593^(1)^	0.010
Gestational weight gain (kg)	13.41 ± 3.09	12.85 ± 2.92	1.682^(1)^	0.095
Pre-pregnancy weight (kg)	62.66 ± 12.12	55.01 ± 10.10	4.849^(1)^	<0.001
Height (cm)	158.65 ± 5.87	159.38 ± 5.46	0.902^(1)^	0.368
Pre-pregnancy BMI (kg/m^2^)	24.86 ± 4.32	21.61 ± 3.28	6.043^(1)^	<0.001
Family history of hypertension			6.244^(2)^	0.012
Yes	19 (23.17)	12 (10.17)		
No	63 (76.83)	106 (89.83)		
Family history of diabetes			10.196^(2)^	0.001
Yes	12 (14.63)	3 (2.54)		
No	70 (85.37)	115 (97.46)		
Pregnancy and Birth	2.60 ± 0.24	2.82 ± 0.31	5.398^(1)^	<0.001
Education level			4.233^(3)^	0.237
Primary school	3 (3.67)	2 (1.69)		
Junior high school/technical secondary school	20 (24.39)	27 (22.88)		
High school/vocational high school	15 (18.29)	12 (10.17)		
College degree and above	44 (53.66)	77 (65.25)		
Fasting blood sugar in early pregnancy (mmol/L)	5.04 ± 0.81	4.53 ± 0.43	5.772^(1)^	<0.001
Total cholesterol (mmol/L)	4.73 ± 1.28	4.56 ± 1.17	0.972^(1)^	0.332
Triglycerides (mmol/L)	2.33 ± 0.45	1.55 ± 0.37	13.408^(1)^	<0.001
HDL cholesterol (mmol/L)	1.30 ± 0.29	1.55 ± 0.40	4.842^(1)^	<0.001
LDL cholesterol (mmol/L)	2.66 ± 0.87	2.54 ± 0.76	1.035^(1)^	0.302

(1) *t*-test; (2) χ2-test; (3) For *Z*-test.

### 3.2 Multifactor analysis of influencing factors related to GDM complicated by HDP

**Figure 2:**
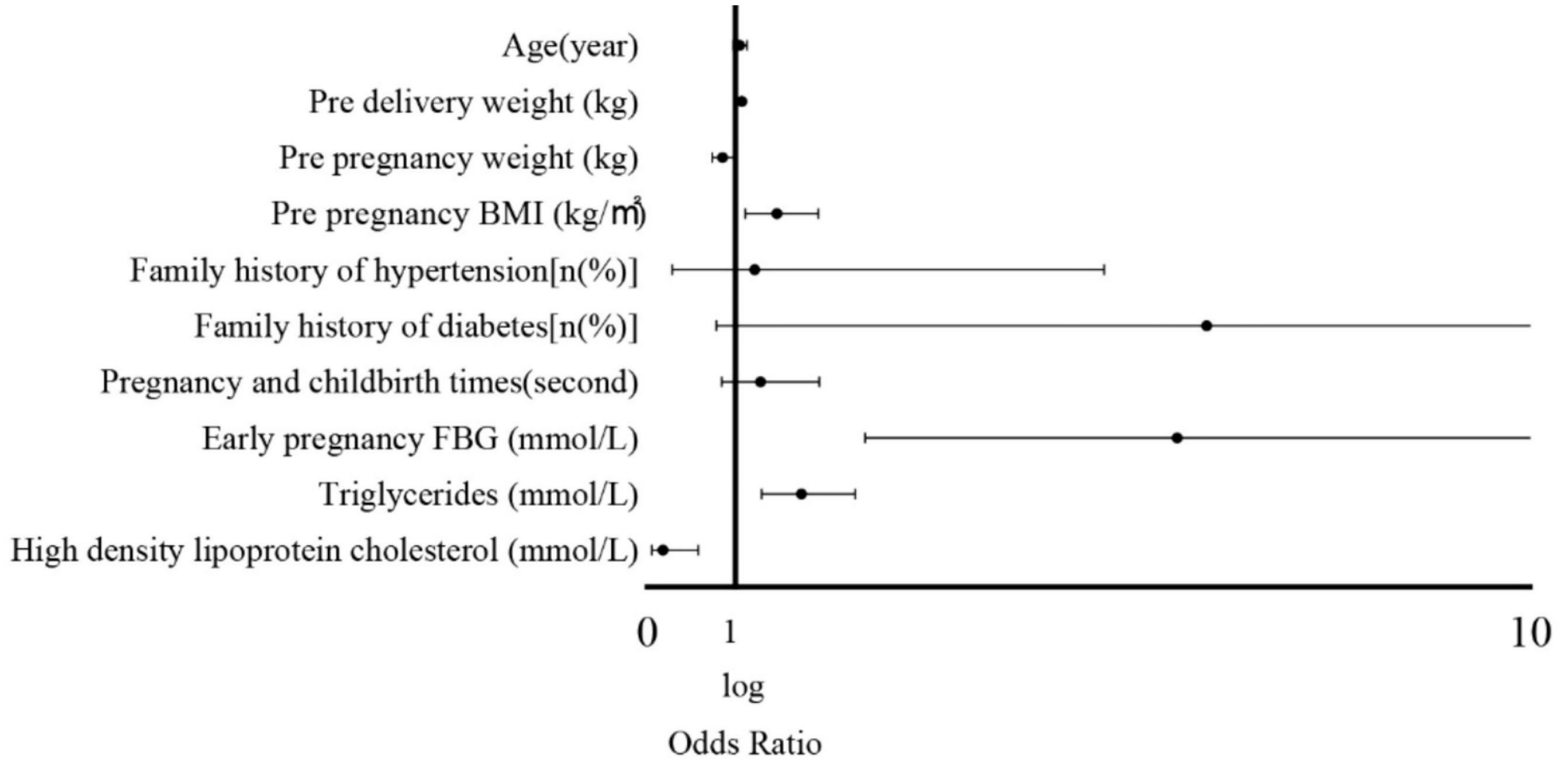
Forest plot of logistic regression analysis of factors influencing adverse pregnancy outcomes.

Univariate logistic regression was first performed to examine the association between each maternal characteristic and adverse pregnancy outcomes (Supplementary Table S1). Variables with *p* < 0.10 or considered clinically significant were then included in the multivariate logistic regression model. The multivariate analysis revealed that pre-pregnancy BMI, early pregnancy fasting blood glucose, triglycerides, high-density lipoprotein cholesterol, and pre-delivery weight were independently associated with the occurrence of GDM complicated by HDP (all *p* < 0.001, Table 2 and Figure 2). These results indicate that maternal anthropometric characteristics and early biochemical markers significantly predict the risk of developing GDM with HDP (Table 3).

**Table 2:** Variable assignment table.

Variables	Variable types	Assignment
Age	Continuous variables	Original value brought in
Weight before delivery	Continuous variables	Original value brought in
Pre-pregnancy weight	Continuous variables	Original value brought in
Preconception BMI	Continuous variables	Original value brought in
Family history of hypertension	Binary variables	have = 1, none = 0
Family history of diabetes	Binary variables	have = 1, none = 0
Pregnancy and Birth	Continuous variables	Original value brought in
Fasting blood sugar in early Pregnancy	Continuous variables	Original value brought in
Triglycerides	Continuous variables	Original value brought in
HDL cholesterol	Continuous variables	Original value brought in

**Table 3:** Logistic regression analysis of factors associated with adverse Pregnancy outcomes.

Variables	*B*	*SEM*	*Ward*	*P*	*Exp(B)*	95% CI	
						Lower	Upper
Age (years)	0.050	0.039	1.644	0.200	1.051	0.974	1.135
Gestational weight gain (kg)	0.070	0.004	306.25	0.000	1.073	1.064	1.081
Pre-pregnancy weight (kg)	−0.155	0.074	4.387	0.036	0.856	0.741	0.990
Height (m)	0.386	0.142	7.389	0.007	1.471	1.114	1.943
Preconception BMI (kg/m^2^)	0.198	0.738	0.072	0.788	1.219	0.287	5.178
Family history of hypertension	1.847	1.066	3.002	0.083	6.341	0.785	51.232
Family history of diabetes	0.251	0.213	1.389	0.239	1.285	0.847	1.951
Fasting blood sugar in early pregnancy	1.793	0.453	15.666	<0.001	6.007	2.472	14.598
Total cholesterol	0.560	0.152	13.573	<0.001	1.751	1.300	2.358
Triglycerides	0.050	0.039	1.644	0.200	1.051	0.974	1.135
HDL cholesterol	−1.720	0.600	8.218	0.004	0.179	0.055	0.580
Constant	−14.076	2.907	2.826	0.093	6.6963E+19	/	/

### 3.3 Comparison of gestational age and delivery mode in GDM combined with HDP

The gestational age at delivery in the GDM combined with HDP group was shorter than that in the HCG (*p* < 0.05). The vaginal delivery in the GDM combined with HDP group was considerably lower than that in the HCG, and the cesarean section was considerably higher than that in the HCG (*p* < 0.05), as depicted in Table 4.

**Table 4:** Comparison of gestational age and mode of delivery in GDM combined with HDP and HCG.

Group	Gestational age at delivery (weeks)	Mode of delivery		
		Vaginal delivery	Late miscarriage	Cesarean section
GDM plus HDP groups (*n* = 82)	38.67 ± 1.49	20 (24.39)	1 (1.22)	61 (74.39)
HCG (*n* = 118)	39.31 ± 1.04	66 (55.93)	0 (0.00)	52 (44.07)
*t/χ^2^*	3.579	20.505		
*P*	<0.001	<0.001		

### 3.4 Comparison of pregnancy outcomes between the GDM with HDP group and the HCG

The total incidence of adverse pregnancy outcomes such as macrosomia, premature rupture of membranes, premature birth, oligohydramnios, postpartum hemorrhage, fetal growth restriction, and pulmonary embolism in the GDM with HDP group was 47.87%, which was considerably higher than that in the HCG (15.35%) (*p* < 0.05, Table 5).

**Table 5:** Comparison of pregnancy outcomes between the GDM combined with HDP group and the HCG.

Group	Macrosomia	Low birth weight	Preterm birth	Oligohydramnios	Premature rupture of membranes	Postpartum hemorrhage	Fetal growth restriction	Pulmonary embolism	Total occurrence
GDM with HDP groups (*n* = 82)	4 (4.88)	0 (0.00)	5 (6.10)	9 (10.98)	11 (13.41)	2 (2.44)	0 (0.00)	1 (1.22)	32 (39.02)
HCG (*n* = 118)	2 (1.69)	1 (0.84)	0 (0.00)	3 (2.54)	11 (9.32)	1 (0.84)	0 (0.00)	0 (0.00)	18 (15.25)
*χ^2^*	/								14.579
*P*	/								<0.001

### 3.5 Blood glucose, blood pressure and adverse pregnancy outcomes in individuals with GDM with HDP after medication treatment

**Figure 3:**
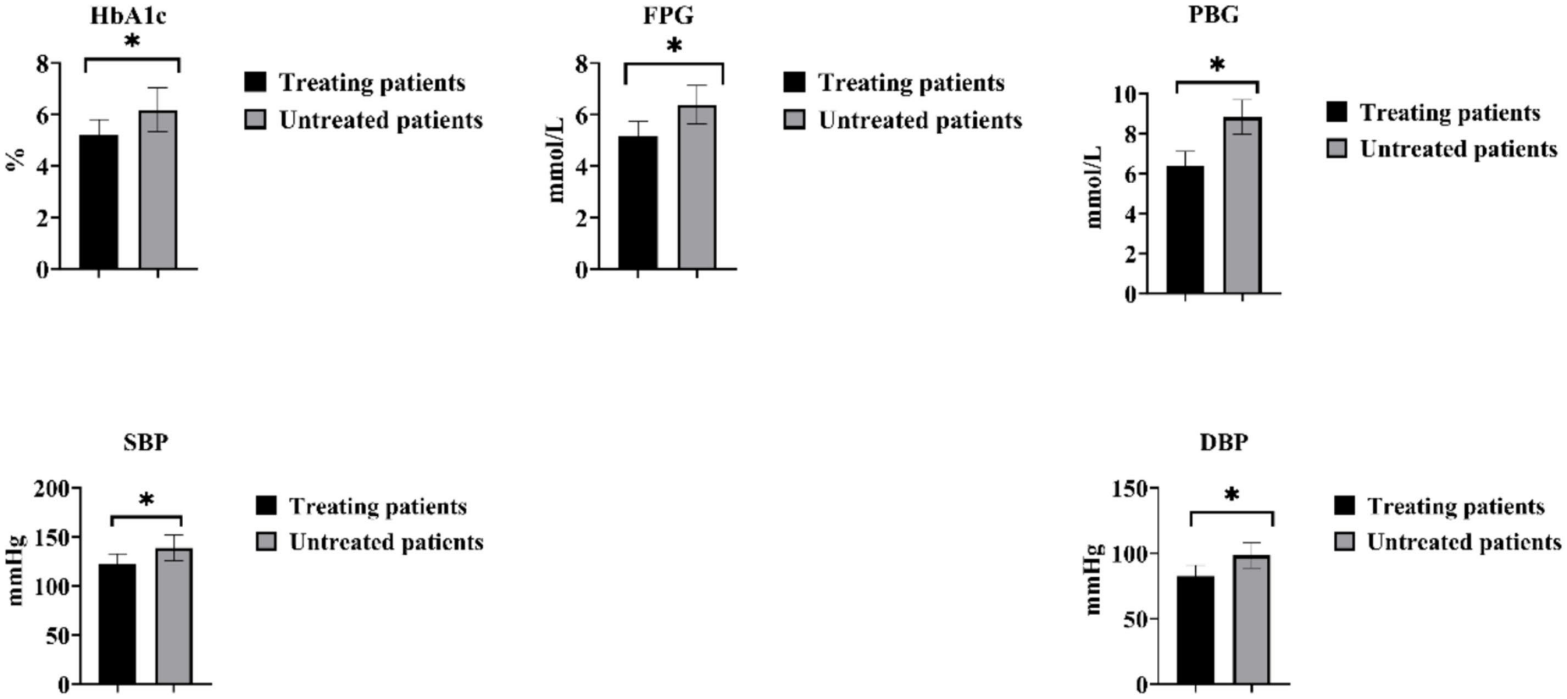
Comparison of blood glucose and blood pressure changes in individuals with GDM and HDP after medication treatment. *p < 0.05.

Among the 82 patients with GDM complicated by HDP, a total of 42 patients (51.22%) received treatment, and all 42 patients underwent antihypertensive therapy. In the treated group, the HbA1c was (5.23 ± 0.57)%, FBG was (5.17 ± 0.58) mmol/L, and 2-h PBG was (6.38 ± 0.72) mmol/L. SBP was (122.38 ± 10.31) mmHg, and DBP was (82.39 ± 8.37) mmHg. In the untreated group, the HbA1c was (6.18 ± 0.85)%, FBG was (6.38 ± 0.75) mmol/L, and 2-h PBG was (8.82 ± 0.87) mmol/L. SBP was (138.98 ± 12.39) mmHg, and DBP was (98.28 ± 9.47) mmHg. The levels of 2-h PBG, FBG, HbA1c, DBP, and SBP were significantly lower in the treated patients compared with the untreated patients (*p* < 0.05), as shown in Figure 3.

The adverse pregnancy outcome rate among untreated GDM patients with HDP was 90.00% (36/40). This included 8 cases (20.00%) of premature rupture of membranes, 5 cases (12.50%) of macrosomia, 8 cases (20.00%) of premature delivery, 8 cases (20.00%) of oligohydramnios, 3 cases (7.50%) of postpartum hemorrhage, 1 case (2.50%) of fetal growth restriction, and 3 cases (7.50%) of pulmonary embolism. In contrast, the adverse pregnancy outcome rate for GDM patients treated for HDP was 21.43% (9/42). This comprised 3 cases (7.14%) of premature rupture of membranes, 3 cases (7.14%) of oligohydramnios, 1 case (2.38%) of fetal growth restriction, 1 case (2.38%) of premature delivery, and 1 case (2.38%) of macrosomia. A significant difference was observed between the treated and untreated groups (χ^2^ = 23.575, *p* < 0.05).

## 4 Discussion

In this study, women with GDM complicated by HDP exhibited significantly higher pre-pregnancy BMI, fasting blood glucose, triglycerides, and HDLC compared with healthy controls. Logistic regression analysis identified pre-pregnancy BMI, early-pregnancy fasting blood glucose, triglycerides, HDLC, and pre-delivery weight as independent predictors for the development of GDM with HDP. These patients also had shorter gestational age, lower rates of vaginal delivery, higher cesarean section rates, and a substantially higher incidence of adverse pregnancy outcomes than healthy controls. Importantly, individualized treatment targeting both glycemic and hypertensive control markedly reduced adverse outcomes, demonstrating the clinical value of early and tailored intervention.

Normal pregnancy is characterized by physiological adaptations such as increased fat intake, enhanced intestinal fat absorption, and elevated hepatic lipid synthesis, contributing to progressive insulin resistance (13). Compared to non-pregnant women, PW show elevated leptin and insulin levels, leading to increased blood lipids and glucose, with lipid levels potentially doubling those of non-pregnant women (14, 15). These changes support fetal growth, maintain pregnancy, facilitate labor, and promote postpartum lactation. However, persistently elevated glucose and lipid levels may result in vascular accumulation, altered blood viscosity, endothelial dysfunction, and systemic inflammation, predisposing to GDM and HDP and increasing maternal and neonatal morbidity (16, 17). In this study, the adverse pregnancy outcome rate among women with GDM treated for HDP was 21.43%. Previous research has indicated that approximately 25% of women with GDM develop HDP (18). The similarity between these figures underscores the high-risk nature of this comorbidity. However, the substantially lower rate of adverse outcomes in our treated cohort compared with untreated patients highlights the protective effect of individualized interventions, suggesting that timely management of blood glucose and blood pressure can partially mitigate the risks associated with this well-documented complication.

Our findings are consistent with previous reports demonstrating that pre-pregnancy BMI, early-pregnancy fasting glucose, triglycerides, and HDLC are key predictors of GDM complicated by HDP (19, 20). In line with these findings, our data further emphasize that the combined burden of dyslipidemia and hyperglycemia before and during early pregnancy substantially heightens the risk of HDP among women with GDM, highlighting a shared pathophysiological pathway. Elevated BMI exacerbates insulin resistance, disrupts lipid metabolism, and promotes inflammatory mediator expression, which can damage vascular endothelium and increase the risk of adverse pregnancy outcomes (21). Similarly, early hyperglycemia reflects underlying *β*-cell dysfunction, which, in combination with pregnancy-induced insulin resistance, accelerates the development of GDM (22, 23). Chronic hypertriglyceridemia further impairs endothelial function, exacerbating hypertensive disorders and supporting the observed pathophysiological synergy between GDM and HDP (24–27). Moreover, this interplay may explain why women with GDM who subsequently develop HDP experience disproportionately higher rates of adverse pregnancy outcomes. These findings underscore the importance of early identification and management of metabolic risk factors before and during pregnancy. Targeted interventions addressing both glycemic and lipid abnormalities may therefore hold promise in reducing the dual burden of GDM and HDP.

The clinical consequences of this dual pathology were evident in our cohort. The GDM + HDP group demonstrated a significantly higher incidence of adverse pregnancy outcomes, including fetal growth restriction, oligohydramnios, macrosomia, premature rupture of membranes, and preterm birth. These findings align with studies highlighting the detrimental effects of hyperglycemia and hypertension on placental perfusion and uteroplacental insufficiency (28–30). The higher rate of cesarean section in this group likely reflects both iatrogenic interventions and maternal-fetal complications. These results underscore the additive burden imposed by GDM and HDP on maternal and fetal health. Early identification of women at risk and timely intervention strategies may mitigate these adverse outcomes. Our study contributes further evidence that integrated management of metabolic and vascular complications is essential for optimizing pregnancy prognosis.

A key observation of this study is the substantial reduction of adverse outcomes with individualized therapy. Targeted management of hyperglycemia and hypertension using insulin, metformin, labetalol, or nifedipine achieved a reduction in adverse outcome rates from 87.8% in untreated patients to 16.98% in those receiving therapy, highlighting the importance of a dual-targeted approach. This finding reinforces prior evidence supporting the efficacy of these interventions in high-risk pregnancies and provides quantitative support for integrated clinical management (31, 32). Importantly, these results suggest that early identification of at-risk women and tailored management strategies can mitigate the synergistic effects of GDM and HDP, providing a framework for optimizing antenatal care.

Several limitations should be acknowledged in this study. First, the single-center design and relatively small sample size may limit the generalizability of the findings. Although pharmacological treatment regimens were closely monitored, inter-individual patient variability could introduce potential bias. Second, the study did not differentiate between specific types of preterm delivery, underscoring the need for more detailed investigations in future research. Finally, the detailed data regarding the specific indications for labor induction were not available. Consequently, we could not perform subgroup analyses to determine whether medically indicated inductions influenced the observed shorter gestational age in the GDM combined with HDP group. Future studies with more comprehensive perinatal records are warranted to assess the impact of labor induction on gestational outcomes in this population.

## 5 Conclusion

In summary, pre-pregnancy weight, pre-pregnancy BMI, HDLC, triglycerides, and early pregnancy FBG are significantly associated with the development of GDM complicated by HDP. These patients are at an increased risk of adverse pregnancy outcomes. Individualized pharmacological management targeting blood glucose and blood pressure effectively mitigates these risks, highlighting the importance of early identification and tailored treatment strategies to improve maternal and neonatal outcomes.

## Data Availability

The raw data supporting the conclusions of this article will be made available by the authors, without undue reservation.
