# Supplementary material for: Clinical features and outcomes of pregnancies complicated by coexisting gestational diabetes and hypertensive disorders
Source: Front Med (Lausanne). 2025 Oct 22;12:1656391. doi: 10.3389/fmed.2025.1656391 (PMC12586028; doi:10.3389/fmed.2025.1656391)
Supplement: Supplementary file 1 [file Table_1.DOCX]

**Table S1**. Univariate logistic regression analysis of factors associated with adverse Pregnancy outcomes.

| **Variable** | **OR** | **95% CI** | **P value** |
| --- | --- | --- | --- |
| Age | 1.04 | 0.98 – 1.11 | 0.18 |
| Gestational weight gain | 1.09 | 1.05 – 1.12 | <0.001 |
| Pre-pregnancy weight | 0.88 | 0.77 – 0.99 | 0.034 |
| Height | 1.45 | 1.12 – 1.88 | 0.005 |
| Preconception BMI | 1.21 | 0.30 – 5.10 | 0.78 |
| Family history of hypertension (yes) | 5.8 | 0.78 – 43.0 | 0.087 |
| Family history of diabetes (yes) | 1.27 | 0.85 – 1.90 | 0.24 |
| Fasting blood sugar in early pregnancy | 5.95 | 2.45 – 14.5 | <0.001 |
| Total cholesterol | 1.72 | 1.28 – 2.32 | <0.001 |
| Triglycerides | 1.06 | 0.97 – 1.16 | 0.18 |
| HDL cholesterol | 0.2 | 0.06 – 0.61 | 0.004 |
